# Supplementary material for: Are carbon emissions trading and green financial instruments synergistic? -Comprehensive quantitative research based on content analysis
Source: PLoS One. 2024 Mar 7;19(3):e0298601. doi: 10.1371/journal.pone.0298601 (PMC10919601; doi:10.1371/journal.pone.0298601)
Supplement: S1 Data — (ZIP) [file pone.0298601.s001.zip › Data/地级市Z_F工作报告.zip!/╣π╬≈/─╧─■╩╨/└┤╫╘╣1⁄2╓┌║┼í╛┬φ┐╦╩2╛▌═°í┐_2014.docx]

各位代表：

　　现在，我代表市人民政府向大会作工作报告，请予审议，并请各位政协委员和列席会议的同志提出意见。

**一、2013年工作回顾**

　　2013年，面对严峻复杂的经济形势和持续下行的经济压力，在自治区党委、政府和市委的坚强领导下，全市各级政府和各族人民坚持稳中求进的工作总基调，以提高经济增长质量和效益为中心，团结奋斗，攻坚克难，全力以赴稳增长、调结构、促发展、惠民生，实现了经济社会平稳较快发展，主要经济指标增速均高于全国、全区平均水平。全年地区生产总值2803.54亿元，增长10.3%；财政收入473.66亿元，增长12.24%；规模以上工业增加值777.52亿元，增长16.6%；全社会固定资产投资2475.01亿元，增长23.4%；社会消费品零售总额1450.84亿元，增长14.01%。实际到位区外境内资金729.23亿元，增长10.28%；直接利用外资（自治区全口径）5.8亿美元，增长15.45%；外贸进出口总额44.2亿美元，增长6.6%。居民消费价格总指数上涨2.1%。被评为“国家节水型城市”“2013中国最佳休闲城市”，实现社会管理综合治理“长安杯”三连冠、“全国科技进步先进市”七连冠，全国城市文明程度指数测评在省会/副省级城市排第八位，位次较2012年提升了9位。

**一年来，我们主要做了以下工作：**

　　（一）调结构促转型，经济质量效益取得新提高。

　　以创新驱动推动产业升级。全市科技财政投入6.21亿元，增长20.43%，高于当年全市经常性财政收入增幅15.58个百分点。专利申请量、专利授权量及发明专利拥有量连续4年位居全区第一，每万人发明专利拥有量增长48.67%。高新技术企业163家，总产值突破860亿元。南宁市科技企业孵化基地被国家科技部认定为国家级科技企业孵化器；世界上直径最大的铝合金圆锭在南南铝试产成功；广发重工集团“中广轨道1号盾构机”正式下线，高端装备制造业实现零的突破。大力实施“工业强市”战略。二次产业比重比上年提高1.24个百分点，电子信息等六大重点产业完成规模以上工业总产值1553.01亿元，增长23.13%，占全市的60.79%，其中，电子信息、铝精深加工规模以上工业总产值增速分别达47.5%、35.4%。工业投资完成728.13亿元，增长19.22%，南南铝等35个投资亿元以上重大项目投产或部分投产，中恒、研祥等82个重大项目开工建设。新增10亿元企业9家、规模以上工业企业28家。全市亿元企业达548家，增加55家，拉动规模以上工业总产值增长22.23个百分点，富士康南宁公司产值157.2亿元，实现百亿元工业企业零的突破。创新开发区管理体制机制，建立奖惩分明、与发展业绩考评挂钩的待遇分配机制，做大做强园区工业，广西—东盟经开区成功升格为国家级开发区，三大国家级开发区规模以上工业总产值占全市比重达47.90%，工业产值超百亿园区6个。淘汰一批小淀粉、小酒精、小造纸等落后产能，万元地区生产总值能耗下降3.5%，降幅达5.22个百分点。大力发展现代服务业。服务业增加值增长8.1%。新增限额以上企业154家，限额以上企业消费品零售总额664.3亿元，增长17.73%。金融业增加值241.11亿元，增长12.4%，占第三产业增加值的17.96%。实现旅游总收入478.15亿元，增长18.39%。举办香港时尚购物展、南宁名品推广周等各类专业会展105场。房地产开发投资416.37亿元，占全区25.79%；商品房销售面积702.6万平方米，增长11.7%。大力支持非公经济发展。民间投资1486.87亿元，增长30.98%，对全市投资增长的贡献率达75.56%。个体工商户24.84万户、私营企业9.23万家，非公企业上缴税收198.2亿元，增长14.43%。

　　（二）强保障促增收，“两个倍增”计划取得新成效。

　　深入实施农民收入倍增计划。全市农业财政投入37.32亿元，增长15.44%，高于当年全市经常性财政收入增幅10.59个百分点。发放农资综合补贴4.5亿元、种粮农民直接补贴2760万元。农林牧渔业总产值578.15亿元，增长4.91%；粮食总产量223.44万吨，再创历史新高，香蕉、茉莉花、木薯、甜玉米产量保持全国第一；新增土地承包经营权流转面积16.2万亩，总量超过100万亩，占全区18.8%。新增市级以上农业龙头企业16家、农民专业合作社200家。建成30个标准化生态养殖基地，新增蔬菜基地7030亩、林下经济7万亩。实施行政村通畅工程，建成通村路384.34公里。完成小（2）型病险水库除险加固138座、农村饮水安全工程495个，解决33万人农村饮水安全问题。完善“三位一体”扶贫大格局，各级财政投入资金2.63亿元，扶持发展特色农产品种植10万亩，修建屯级道路410条487.74公里、桥梁14座，解决29万名群众行路难问题。新增农村劳动力转移就业9.9万人。农村居民人均纯收入7685元，增长13.4%。深入实施城镇居民收入倍增计划。企业退休人员基本养老金实现“九连涨”，人均达1621.23元/月；最低工资标准提高至1200元/月，增幅20%。加强就业援助，累计发放小额担保贷款3.38亿元。城镇新增就业8.6万人，城镇登记失业率3.01%。城镇居民人均可支配收入24817元，增长10%。

　　（三）重招商优服务，以投资促增长取得新佳绩。

　　紧盯世界500强、全国知名民营企业，市四家班子领导率团赴粤、港、澳、台等地区开展招商活动，抓住全国工商联执委会在邕举办的机遇，全力以赴招大商引强企，成功引进绿地集团等6家世界500强企业，宝能、万科、研祥、海王等国内20多家金融、地产、电子、医药等行业龙头企业落户南宁。加强投资软硬环境建设，南宁投资环境满意度排名全区首位。建立市领导联系服务重大项目和重点企业新机制，重点领域、重大项目建设取得新进展，完成基本建设投资1109.74亿元、增长29.16%，更新改造投资728.33亿元、增长23.08%；自治区、市层面统筹推进重大项目559项，完成投资743.48亿元；城建项目477项，完成投资220.9亿元，增长16.44%。

　　（四）抓改革促开放，发展动力取得新增强。

　　深化重点领域改革。积极推进统筹城乡改革。出台《南宁市农村住宅建设管理及推荐户型奖励办法》，重点推进坛板坡等12个综合示范村建设。首次将政府性基金、社保基金纳入全口径预算，首次将社保基金预算、国有资本经营预算纳入同级人大审议。将28家国有独资及国有控股企业，按行业、资源整合重组为九大集团（公司）。深化行政审批制度改革，授予三大国家级开发区管委会105项行政许可项目、127项非行政许可审批项目；将8大类89项行政审批、行政处罚等管理事项委托或授权五象新区管委会，市政务服务中心进驻五象新区，实现五象新区项目审批“不过江”目标；强化行政审批职能、事项、人员“三集中”，全市279项行政审批事项再次提速，承诺提速率平均达50%以上。积极推进事业单位分类改革。全面启动六县县级公立医院综合改革；基层医疗卫生机构综合改革基本完成，88.44%的政府投资建设村卫生室纳入新农合定点医疗机构范围。提升对外开放水平。成功服务第十届中国—东盟博览会和商务与投资峰会、中国—东南亚民间高端对话会议、第二届中越青年大联欢活动等。进一步推进内地与香港、澳门更紧密经贸关系安排（CEPA）示范城市建设。鼓励企业“走出去”，全市申报境外投资企业18家，投资总额4.32亿美元，增长58%。

　　（五）抓建设优环境，城市宜居水平取得新提升。

　　突出五象新区开发建设。新区管委会挂牌成立，全面推行“一线决策、一线审批、一线协调”工作法，项目开工不断创造新的“五象速度”。统筹做好市政基础设施、公共与配套设施等专项规划，重点片区规划进一步完善。积极探索区域连片土地统征统储和集中安置方式，开工建设12个安置点，妥善解决1.3万人安置问题。落实新增建设用地指标、“招拍挂”出让土地面积、征地完成量分别占市本级的44%、40%、50%。实施项目130项，完成投资153.62亿元。广州恒大等30多家国际、国内知名企业落户五象新区，兴业银行、太平保险等9家金融、保险机构集聚金融街，总部基地95%以上的土地已落实项目，投资达500多亿元。加快重大交通基础设施建设。轨道交通1号线完成投资32.58亿元，2号线顺利开工，轨道交通第二轮规划（含3、4、5号线）已报国家发改委审批。南桂、南柳及南宁至钦北防高铁相继开通，市辖区内高铁里程306.27公里，南宁迈入高铁时代。南宁吴圩国际机场新航站楼主体完工，火车东站及周边路网建设全面推进。城市道路“白改黑”工程完成224条。良庆大桥开工建设，白沙大道3座立交桥、金湖广场下穿隧道等工程如期建成通车。经强力攻坚，历经9年建设的民主铁路立交桥、凌铁大桥建成通车，历经8年建设的长堽路打通至快环。扎实推进旧城改造。完成投资约51.7亿元，水街片区旧城区改建、老南宁·三街两巷旧城改造项目顺利推进，完成新阳路292号等20个土地熟化项目。重拳整治“两违”，拆除违法建设面积381.4万平方米、清理违法占地面积506.5万平方米。抓好“中国水城”建设。完成投资50.05亿元，增长81.1%。邕江两岸综合整治示范段（3.4公里）、良庆河综合整治工程三期如期建成，邕宁水利枢纽、江北引水干渠开工建设，老口水利枢纽完成投资13.4亿元，民歌湖、相思湖、明月湖等环城水系进一步提升。提升“中国绿城”水平。完成投资19.5亿元，增长34.96%。完成新造林24.49万亩，村屯绿化130个。成功举办第三届广西园林园艺博览会，五象湖公园成为新亮点。民族大道、五象大道、竹溪大道等城市精品线路的园林景观进一步提升，城市绿化、美化、彩化三年行动计划成效凸显。全市建成区绿地率、绿化覆盖率和人均公园绿地面积分别提高0.3、0.23个百分点和0.21平方米。环境空气质量（AQI指数）优良率达75%，在省会城市（直辖市）中名列第六。

　　（六）办实事惠民生，社会事业发展取得新进步。

　　全市财政涉及民生支出292.74亿元，比上年增支33.58亿元，占全市财政支出的69.87%。完善社会保障体系。全市社会保险参保608.28万总人次（不含新农合）。投入3.4亿元提高城乡最低生活保障标准和五保户、孤儿供养标准，覆盖城乡居民248.48万人次。安排社区惠民资金3520万元，解决一批社区居民最关心的民生问题。开工建设各类保障房19900套、基本建成17856套，新增发放廉租住房租赁补贴2568户、分配入住保障性住房6295套。全面发展教育事业。教育支出70.32亿元，增长6.43%，高于当年全市经常性财政收入增幅1.58个百分点。农村义务教育学生营养改善计划安排2.33亿元，受益学生40万人，率先在全区实现市辖县所有义务教育阶段学生全覆盖。完成幼儿园建设项目386个，创建自治区级示范幼儿园6所、市级示范幼儿园37所，超额完成学前教育三年行动计划。建成12所城市中小学校，接收进城务工人员随迁子女义务教育学生12.6万人，占全区33.18%。学前三年毛入园率、九年义务教育巩固率、高中阶段毛入学率分别提高4.31、0.56、0.62个百分点。加快发展卫生事业。新型农村合作医疗参合率达99.38%；726万人次参合农民获医疗费用补偿，补偿基金19亿元，基金使用率111%。人均基本公共卫生服务经费标准提高5元。全力实施防治艾滋病攻坚工程、母婴健康“一免二补”幸福工程、地中海贫血防治计划、中医药民族医药重点工程和壮瑶医药振兴计划，全市社区卫生服务中心、乡镇卫生院实现中医科全覆盖。全市人口自然增长率8.37‰，出生人口性别比同比下降2.75个比值。大力提升文化软实力。市财政投入4500万元完成129个村级公共服务中心建设。成功打造“一声所爱·大地飞歌”2013新民歌音乐季，汪小敏等3位选手成功入选央视春晚。《天琴传说》获全国曲艺优秀节目展演优秀剧目奖，实现南宁曲艺全国夺奖零的突破。南宁电视台与6家境外媒体成功举办跨国春晚《春天的旋律》，节目信号覆盖泰国等东南亚10国及北美、欧洲、大洋洲等地区。安排5000万元专项扶持文化产业发展，中国—东盟创意乐园（锦园）项目落地，天天演项目《锦·宴》对外公演。村村通广播电视乡镇无线覆盖新增25万人。保护和弘扬壮族文化遗产，武鸣县获“中国歌圩文化之乡”称号。推进体育事业发展。全市经常参加体育锻炼人口达49%。成功举办2013南宁国际半程马拉松比赛、南宁国际龙舟赛等5项国际体育赛事。高标准做好第四十五届世界体操锦标赛筹备工作。全力稳定物价。建立社会救助和保障标准与物价上涨联动挂钩机制，开通菜篮子工程平价直销车20辆、设立农副产品平价商店26家，实行“一元菜”供应。实施为民办实事工程。投入58.12亿元，自治区10项、南宁市20项为民办实事工程104个子项完成100项，4个子项正在收尾，解决了一大批群众关心的热点难点问题。

　　（七）抓规范严管理，社会和谐稳定取得新成效。

　　推进“平安南宁”建设，新增3000个网络高清监控摄像头，严厉打击“两抢一盗”，案件发案数和刑事案件发案数分别下降15.98%、23.05%。狠抓安全生产工作，年内无重大以上安全生产事故，年度考核综合排名全区第一。开展乳制品等重点食品、餐厨废弃物及废弃油脂等十大专项整治行动，市、县（区）、乡镇（街道）食品药品监管体系改革工作走在全区前列。“智慧南宁”建设成为国家试点。全国民族团结进步示范市试点、全国少数民族流动人口服务管理试点工作扎实推进。平里静脉产业园开工建设，餐厨废弃物资源化利用和无害化处理厂基本竣工，城市污水截污及管网建设实现新突破，市本级生活污水集中处理率、生活垃圾无害化处理率分别达85%和100%，邕江地表水集中式饮用水源水质达标率100%。强力推进“美丽南宁·清洁乡村”活动，打造示范点138个，乡村脏乱差现象得到明显改善。大力开展“美丽南宁·整洁畅通有序大行动”，营造了整洁畅通、文明有序的城市环境，实现了“100天新变化、200天新提升”的工作目标，得到了全市人民的高度评价。

　　（八）改作风提效能，政府自身建设取得新加强。

　　加强政府立法，完善交通、食品安全、信用体系、违法建设查处等重点领域的制度建设。推进依法行政，严格按程序起草、审议、发布文件，提请市人大常委会审议地方性法规草案5件，出台政府规章4件、废止3件。主动公开政府信息11.7万多条，信息公开量继续保持全区首位。推进“一服务两公开”向基层延伸，98.4%的乡镇（街道）和43.9%的行政村（社区）建成政务服务中心，超额完成自治区下达的任务。认真贯彻落实中央“八项规定”精神，全市会议召开数量同比下降31.63%；处级干部因公出国（境）人数下降35.01%，科级干部因公出国（境）人数下降52%；公务接待费用下降24.95%，公务用车运行经费下降7.2%，节庆、论坛活动经费下降61.9%；腾退办公用房1380间、40887平方米，清理、停止楼堂馆所建设项目21个。积极开展信访工作，接听“市长公开电话”来电4.03万个，受理“人民网市长信箱”留言618条，妥善处理群众来信3234件、来访2378批9030人次。强化对政府投资项目、重大政策执行等审计监督，廉政建设进一步加强。认真执行市人大及其常委会的决议、决定，依法接受市人大及其常委会监督，自觉接受人民政协的民主监督和社会舆论监督，共办理自治区和市级人大代表议案建议261件、政协委员提案338件，办结率100%。认真听取各民主党派、工商联、无党派、人民团体和社会各界人士的意见。

　　一年来，我市外事、侨务、司法、统计、人防、保密、口岸、水库移民、地震、供销、农机、机关事务管理、地方志、档案、宗教、新闻出版、消防、海关、海事、检验检疫、税务、工商、质监、国家统计南宁调查队、通信、邮政、供电、测绘、气象、水文、参事、哲学和社会科学等工作取得新进展，妇女儿童等事业取得新进步。国防教育和后备力量建设深入开展，双拥共建工作取得新的成绩，少数民族聚居区繁荣发展。

　　各位代表，过去的一年，在困难和压力多年来少有的情况下，我们顶住了压力，经受住了考验，取得了来之不易的成绩。这是自治区党委、政府坚强领导的结果，是市委总揽全局、科学决策的结果，是市人大及其常委会依法监督和人民政协民主监督的结果，也是全市各族人民共同奋斗的结果。在此，我代表市人民政府，向辛勤工作在各条战线的工人、农民、知识分子、干部和社会各界人士，向驻邕人民解放军、武警部队官兵，向各民主党派、工商联、无党派人士、人民团体，向关心支持南宁发展建设的港澳台同胞、海外侨胞及海内外朋友们，表示崇高的敬意和衷心的感谢！

　　同时，我们也清醒地看到，我市构建现代产业体系，做大经济总量的任务十分繁重；科技带动能力较弱，创新驱动发展亟待加强；影响科学发展的体制机制瓶颈依然不少，改革攻坚面临重重挑战；城乡发展不平衡仍然突出，新型城镇化建设任重道远；社会和谐稳定压力依然存在，社会治理仍需进一步加强；政府自身建设、干部作风有待进一步加强和改进等。对这些困难和问题，我们不掩饰、不回避，必须高度重视，切实加以解决，决不辜负全市各族人民的期望！

**二、2014年工作安排**

　　2014年，是深入贯彻落实党的十八届三中全会精神、全面深化改革的第一年，是完成“十二五”规划的关键一年。自治区党委十届四次全会明确提出了“两个建成”的奋斗目标。市委十一届十一次全会作出了全面深化改革奋力提升南宁首位度的决定，提出力争两年打基础、三年大跨越，用五年左右的时间抓改革、促发展、惠民生，实现南宁首位度大提升的目标。

　　各位代表，错综复杂的国内外形势提醒我们，扎实做好2014年经济社会发展工作，决不能把发展简化为生产总值，只盯住眼前的增长速度，而是要统筹全局，稳中求进，用改革的精神、思路、办法，激发市场活力，释放发展的强大动力。我们坚信，在市委的正确领导下，经过全市各族人民的共同努力，市委确定的奋斗目标一定能够实现，首府南宁在全区经济社会发展中的首位度一定会得到大提升！

　　2014年政府工作的总体要求是：全面贯彻落实党的十八大、十八届二中和三中全会、中央经济工作会议、中央城镇化工作会议、中央农村工作会议以及自治区党委十届四次全会、市委十一届十一次全会精神，以抓改革、促发展、惠民生为主基调，始终保持高于全区平均水平的发展速度，突出全面深化改革、产业转型升级、新型城镇化建设、保障和改善民生，进一步扩大开放合作，举全市之力办好第四十五届世界体操锦标赛，奋力提升首府南宁在广西经济社会发展中的首位度，加快建设中国面向东盟开放合作的区域性国际城市、宜居的壮乡首府和具有亚热带风情的生态园林城市。

　　按照上述总体要求，2014年经济社会发展的主要预期目标为：地区生产总值增长10%；财政收入增长10%；全社会固定资产投资增长18%；规模以上工业增加值增长16%；社会消费品零售总额增长14%；外贸进出口总额增长8%；城镇居民人均可支配收入增长10%，农村居民人均纯收入增长12%；居民消费价格总指数涨幅控制在4%左右；万元生产总值能耗、化学需氧量排放量、二氧化硫排放量、氨氮排放量、氮氧化物排放量按自治区下达的目标完成；城镇化率59.2%；每万人有效发明专利拥有量2.5件；学前三年毛入园率91%，九年义务教育巩固率93%，高中阶段毛入学率92%；城镇新增就业人数7.5万人，城镇登记失业率控制在4.5%以内。

　　各位代表，实现上述目标，有机遇、有条件、有潜力，但也有挑战、有难度、有压力，我们必须坚定信心，攻坚克难，努力工作，争取更好更快的发展。

　　（一）以改革创新为统领，推动发展活力新增强。

　　深化行政审批制度改革。做好行政审批事项“接、放、管”，市本级的行政审批事项，能减就减，能放就放，能取消就取消，坚决控制新增行政审批事项，对保留事项依法管好。创新和完善重大项目联合审批、预约审批、缺项审批、分段审批等审批方式，探索建立“一门受理、抄告相关、同步审批、限时完成”审批模式，推行“网上受理、网上流转、网上审批、政务中心出件”审批流程，解决部门和政务中心两头受理问题。加快建立网上政务服务大厅，实现所有审批项目的实时公示。深化政府机构改革，推进事业单位分类改革。打造公平、开放、便捷的发展环境。

　　深化财政体制改革。构建和完善覆盖政府所有收支的全口径预算体系。继续加强预算绩效管理，扩大绩效目标管理和绩效考核覆盖面。实施规范、公开、透明的预算制度，稳步推进财政预决算公开和“三公”经费公开。探索编制全市各级政府性债务收支计划，加强政府债务管理。完善市区事权和支出责任划分，创新市本级与城区财政利益共享机制。整合各类产业发展资金，建立健全财政促进产业发展机制。建立健全政府购买服务的长效机制。让预算更加透明，支出更加规范，公共财政更好地为民服务。

　　深化土地管理制度改革。完善城乡建设用地增减挂钩制度，试点推进农村集体经营性建设用地使用权流转、城市低效土地开发利用等改革。加快农村集体土地和宅基地确权登记发证工作，在符合土地利用总体规划和土地用途管制前提下，允许农村集体经营性建设用地通过出让、租赁、入股等方式依法进行流转，建立完善土地租赁、转让、抵押二级市场，逐步将农村集体经营性建设用地使用权、林地经营权和林木所有权、土地承包经营权等纳入城乡统一的土地交易市场。完善被征地农民合理、规范、多元的保障机制。确保土地利用更加集约高效，土地收益分配更加公平。

　　深化城镇化发展体制机制改革。改革户籍管理制度，有序推进农业转移人口市民化。创建多元可持续的城镇建设资金保障机制，争取发行市政建设债券，更多地吸引社会资本参与城市公用设施投资、建设、运营。健全城市基础设施服务合理收费机制。完善城市规划制定、实施、监督的地方性法规，强化规划指导和约束。制定出台促进房地产市场持续健康发展政策。破除城镇化发展体制机制障碍，让广大农民平等参与城镇化进程，共享现代化成果。

　　深化国有企业改革。完善以管资本为主的国有资产管理体制，重点加大九大集团（公司）改革力度，竞争类的国企加快推向市场，将国资重点集中在基础设施和民生保障等关键领域。深化国有企业公司制、股份制改革，大力发展混合所有制经济，支持和鼓励各种非公资本参与国企股权多元化改革。探索建立国有企业分类监管制度，健全国有企业领导人员管理体制。释放国有企业发展的活力、创造力和竞争力。

　　大力发展非公经济。坚决废除对非公经济各种形式的不合理规定，进一步清除市场准入障碍，消除各种隐性壁垒，出台非公企业进入特许经营领域具体办法。完善财税支持、产业扶持、政府服务等配套措施，市财政每年统筹安排不低于5000万元支持非公经济发展，重点培育100家以上非公强优企业。引导非公企业兴办文化、医疗、养老等社会事业。让南宁成为非公企业投资、创业的沃土。

　　（二）以“工业强市”战略为牵引，推动现代工业大发展。

　　以科技创新引领工业发展。大力支持富士康南宁科技园研发检测认证中心建设和研祥、燎旺车灯等一批骨干企业创建国家级技术中心、实验室。扶持培育高新技术企业15家、创新型试点企业10家。重点培育20个广西名牌产品和广西著名商标，认定工业新产品100个以上。重点推进智能制造、网络制造、工业大数据集成为重点的“两化”融合项目建设，力争完成制糖、铝精深加工、机械装备、食品药品“两化”融合方案。投资10亿元，完成技术创新及“两化”融合项目300项。

　　以重点产业发展优化工业结构。新增建设用地指标40%以上用于工业项目及工业园区基础设施建设，专项安排16.5亿元现代工业发展资金，集中支持生物医药、铝精深加工、电子信息、机械装备制造、轻工食品、清洁能源六大重点产业发展，力争实现工业总产值1850亿元、税金115亿元以上。以生物医药产业园为平台，重点推进中恒、海王等项目建设。以南南铝为龙头，重点加强与中国南车等集团合作，全面发展铝精深加工。加快与知名汽车企业对接，努力在汽车整车制造项目上取得新突破。加快建设南宁电子信息产业园，力争富士康南宁公司实现产值200亿元。重点支持南宁双汇食品、百威啤酒年产30万吨啤酒生产线等项目建设，将广西—东盟经开区打造成为全区重要的轻工食品生产基地。大力发展生物质能源，努力将武鸣县建设成为全国生物燃气重点县。坚持“壮大关小”，坚决淘汰小造纸、小淀粉、小酒精等落后产能。

　　以做大园区聚合工业优势。在各工业园区实施“体制机制创新”、“企业培育”、“产业提升”、“零增地技术改造”、“科技创新”、“金融创新”、“人才引进”、“基础设施建设攻坚”、“项目引进建设攻坚”九大行动计划，重点推进150万平方米工业标准厂房建设，不断完善园区基础设施，提高承载大项目能力。将国家级开发区改革创新发展政策逐步延伸到县区工业园区（集中区）。高标准规划建设南宁吴圩空港经济区，加快新兴产业园、现代工业园规划建设及招商。继续支持三大国家级开发区管理体制机制创新和产业发展，确保规模以上工业总产值占全市的比重提高5个百分点以上。

　　以优质服务做强工业企业。继续实施领导联系服务重大项目和重点企业工作机制，协调解决企业在融资、用工、用地等方面的问题和困难。发挥“两台一会”作用，帮助中小企业实现融资30亿元以上。鼓励和支持企业股权融资，引导具备条件的企业进行股份制改造。积极争取自治区支持，成立北部湾经济区（南宁）产业发展基金，争取实现融资100亿元。继续实施“抓大壮小扶微”工程，加快推进广发重工重组及改造、凤凰纸业和华劲纸业异地搬迁等重大项目，支持中烟南宁卷烟厂产值突破100亿元，实现全市百亿元企业新增1家、10亿元以上企业新增3家、亿元以上企业新增50家、规模以上企业新增25家的目标。

　　（三）以金融业为核心，推动现代服务业发展。

　　加快金融业发展。抓住滇桂沿边金融综合改革试验区建设机遇，积极发挥金融发展决策咨询委员会和金融工作联席会议制度作用，制定更加优惠的政策，营造更好的金融生态环境，利用好5000万元金融产业发展资金，吸引国内外各类金融机构及会计、审计、财务等金融中介机构进驻南宁，争取正在筹建的亚洲基础设施投资银行、中国—东盟股权交易中心和互联互通投融资平台落户南宁，大力推进五象新区金融街等项目建设，加快南宁区域性金融中心建设。完善扶持企业上市相关规定，推动南城百货等4家企业上市。力争金融业增加值增长15%以上。

　　推动旅游业发展。坚持政府主导，突出市场主体，打造“壮乡歌海、中国绿城、东盟风情、养生之都”四大品牌，把旅游产业培育成战略性支柱产业。加大旅游宣传和市场营销，加强旅游基础设施建设和旅游产品开发，打造旅游精品线路。大力推进上林县特色旅游名县创建和双桥镇、布泉乡等10个南宁特色旅游名镇（乡）建设；重点建设东盟国际旅游风情小镇等28个重大旅游项目。加快青秀山风景区植物专类园建设和大明山旅游景区建设。力争创建国家5A级旅游景区1家、4A级旅游景区3家，旅游总收入突破550亿元。

　　发展现代商贸物流业。加快完善商贸流通规划，合理布局城市商业综合体、特色商业街、专业市场、社区商业中心、乡镇商贸中心等商业流通设施及业态，改造朝阳商圈，完善埌东—凤岭商圈，规划建设五象商圈。重点推进万达、宝能等城市综合体建设，力争新增限额以上企业100家。优化物流园区布局，大力支持集智能仓储、现代物流、金融服务于一体的一站式第三方大宗商品供应链服务平台建设，大力发展综合物流中心、专业物流中心和配送中心，推进中国—东盟商品交易中心、中国—东盟国际物流基地等重大项目建设，加快建成南宁综合保税区。

　　发展楼宇经济。编制楼宇经济发展规划，研究出台扶持政策，创新政府服务模式，大力发展资金、技术、服务、税收密集的楼宇经济，重点培育一批特色街区楼宇、现代服务型楼宇、文化创意型楼宇、总部型楼宇等高附加值楼宇，为进一步推动我市产业升级、经济发展注入新的动力。

　　发展电子商务。以建设面向东盟区域性电商总部和集散中心为目标，推进国家电子商务示范城市建设。强化政策支持，鼓励美丽传说、蓝火翼网络科技等有条件的电子商务企业探索智能物流、跨境电子商务等新型业务，争取阿里巴巴等国内电商龙头企业落户南宁。加快高新区国家电子商务示范基地建设。

　　（四）以农业现代化为主导，推动“三农”持续发展。

　　推进农业结构优化升级。贯彻落实中央一号文件精神，实施“粮安”工程，划定基本农田，确保粮食总产量220万吨，农产品质量安全监测总体合格率96%以上。强化农业科技支撑，加强标准化农田和规模养殖场建设，推进农业标准化生产，加快发展优势特色种植业，积极推广禽畜生态循环养殖，大力发展林下经济，提高农产品深加工能力。建设优势特色产业示范基地30个、“菜篮子”基地4000亩。

　　完善农业发展新机制。积极发展农村社区股份合作社、土地股份合作社，继续推广“朝南模式”、“金穗模式”，帮助农民在“家门口”增产增收。重点扶持桂合丝业、百洋集团等龙头企业发展，新增10家市级以上农业产业化龙头企业。大力培育专业大户、家庭农场、联营农户等新型农业经营主体，扶持发展30个以上“一村一品”、“一镇一业”特色产业，促进农民收入倍增计划实施。

　　推进农业社会化服务。支持新型农业经营组织围绕产前、产中、产后各环节为农户提供全程服务。加大农业政策性保险支持力度，完善基层农技推广体系，深化公益性服务能力建设，加快发展以农作物病虫害统防统治、农机社会化服务等为重点的农业经营性服务。加强农产品网络展销平台建设，扶持农超、农校、农企对接。

　　加强农村基础设施建设。继续抓好人饮工程，解决30万人饮水安全问题。继续实施小型病险水库除险加固120座，推进小型农田水利重点县建设，改善和新增灌溉面积7.5万亩，着力解决农田灌溉“最后一公里”问题。新建200条非贫困村通屯道路。继续实施行政村通畅工程，实现97%以上的行政村通水泥路。加强防灾减灾体系建设，确保农业可持续发展。

　　（五）以五象新区建设为重点，推动投资消费平稳较快增长。

　　全力推进投资和项目建设。创新模式，探索通过存量资产、土地使用权作价注资、项目资产、财政专项资金注入各投融资平台公司，不断增强融资能力。优化投资结构，鼓励引导资金投向先进制造业、战略性新兴产业、现代服务业、生态环保等领域。落实自治区、市级统筹推进重大项目556项、投资740亿元；推进城建计划360项、投资340亿元。高档次高品质推进五象新区建设。支持新区解放思想、大胆创新、先行先试。集中精力突破核心区域，确保年内总部基地的项目全面开工，抓好五象湖周边及沿江区域189个重点项目建设，力争完成投资275亿元以上。加快跨江桥梁及蟠龙、玉洞、龙岗、保税物流等重点片区基础设施建设。加大管廊规划和建设力度，完善给排水、电力电讯、燃气、环卫等市政配套设施，全面启动新区学校、医院、邻里中心等城市配套建设。大力推进征地拆迁和安置房建设，全面破解被征地拆迁农民安置工作滞后问题。力争3—5年，把五象新区建设成财富聚集、功能多元、生态宜居、辐射强劲的高品位魅力新城以及我市城市建设和产业发展先行先试的试验区。大力推进重大交通基础设施建设。实施项目47项、投资225亿元以上。建成南宁吴圩国际机场新航站楼及机场高速公路连接线、火车东站及周边路网。加快轨道交通1、2号线建设，力争3号线开工，完成投资50亿元以上。加速云桂、南广等高铁项目建设，加快外环高速、南北高速改扩建及来宾至马山、马山至平果等高速公路建设，推进新江至扶绥、大王滩一级路等高等级公路建设。加快推进南宁港一期工程建设，开工建设南宁港永凯码头、鹤笋作业区一期、横县国铭码头工程等。

　　着力扩大消费需求。继续开展“南宁欢乐消费季”等多种形式的促销活动，加快推进传统零售业转型升级，促进线上线下深度融合，扩大汽车、家电、通讯器材等传统消费。以建设国家信息消费试点城市为契机，培育信息、文化、健康、养老等新型消费热点，推动消费结构升级。加快发展大众化、特色化餐饮消费，促进中低端消费成为餐饮消费增长的主动力。加快推进社区商业中心、乡镇商贸中心等仍具有较大潜力和发展空间的业态发展。

（六）以精细化管理为手段，推动生态宜居城市建设。

　　提升“美丽南宁”建设水平。持续推进“美丽南宁·清洁乡村”活动，深入发动群众，继续实施“三清洁”综合整治等五大行动，启动“美丽南宁·生态乡村”活动，推进村屯绿化、饮水净化、道路硬化，改善农村人居环境。调整优化村庄布局，大力推广并落实农村住宅建设及推荐户型奖励办法，形成美丽村庄新格局。在12个县区各建成一个综合示范村，创建3个生态乡镇、100个生态村。深入开展“美丽南宁·整洁畅通有序大行动”，加快在城管、建设、国土、住房、环保和文化等部门推进相对集中行政处罚权改革，在市政养护、环卫作业、小广告清理和绿化养护等领域开展市场化社会化改革试点，加强城市户外广告规划管理；继续实施严管重罚，坚决查处城市“五乱”行为；实施“公交优先”战略，加快调度中心、公交场站、公交专用道等城市公交基础设施建设，提升公交管理科学化水平；大力推进“城市网格化管理决策服务平台”建设，实现网格化管理智能化及城市管理实时监测和快速反应，提高交通组织管理科学化水平，治理城市交通拥堵和停车难问题，实现“500天新形象”工作目标。

　　推进城市绿化美化彩化。按照生态自然化、森林化、多彩化、多样化、苗木本土化、管理低成本化原则，全面提升城市绿化美化彩化水平。提升“绿城”，重点推进城市精品线路园林景观提升、城市立交景观提升等八大工程；改造提升市政主干道、景观节点30条（处），重点推进五象大道、白沙大道等17条城市主干道绿化提升，加快实施白沙—星光立交桥等6座立交桥和13座人行天桥园林景观改造；建设和提升江南公园、花卉公园等公园。重点推进青秀山风景区绿道、快速环道绿道（竹溪立交—壮锦大道段）及景观提升工程，新建绿道45公里，建成区新增绿地面积400公顷，改造提升绿地200公顷。建设“水城”，继续推进江北引水干渠主体工程；加快南湖等“五湖”环湖水系景观提升和相思湖、明月湖水系修复；续建老口水利枢纽和邕宁水利枢纽。加快推进污水管网建设三年行动计划，建设污水管网250公里。加快大王滩第二水源建设，继续推进天雹水库、龙潭水库等应急水源建设，支持宾阳县清平水库补水工程、马山县兴科供水工程建设，提升城镇安全供水保障能力。加快仙葫半岛堤、龟山堤、隆安县和横县县城防洪堤建设。推动全国水生态文明城市试点工作。打造“花城”，全面推行盆花下地培土种植，适度缩减草花栽植面积，选用多年生、花期长、花色艳、易维护的花卉，千方百计降低养护成本，精心营造四季花城的景观效果，确保一年显成效、两年大提升。

　　加快“三旧”改造。以创建“国家历史文化名城”为抓手，坚持“政府主导、城区主体、整体规划、连片开发”的原则，继续抓好水街片区旧城区改建、老南宁·三街两巷旧城改造项目。将城中村改造、危旧住宅片区项目纳入棚户区改造计划，确保陈东村、南糖片区、四塘煤矿片区等10个项目取得新突破。

　　（七）以新型城镇化为引擎，推动城乡一体化发展。

　　加快新型城镇化建设。坚持“以人为本、优化提质、产城一体、统筹发展、改革创新”的原则，加快城乡一体化发展。突出大县城战略，把发展中小城市、小城镇作为主攻方向，编制南宁市空间发展战略规划，加快六县县城和重点乡镇规划修编，构建大中小城市和小城镇合理分布、功能互补、协同发展的城镇体系，努力将南宁建设成为北部湾城市群的核心城市。按照县级中等城市的要求，加快推进武鸣县、横县、宾阳县规划建设，力争到2015年，三县城镇化率40%以上。按照小城市的要求，加快上林县、马山县、隆安县规划建设，力争到2015年，三县城镇化率30%以上。加快双桥、六景、黎塘等20个经济强镇、特色城镇建设。坚持产城融合发展，把产业发展摆在新型城镇化建设的首位，以产业集聚带动人口集聚，以产业发展带动城镇转型升级。

　　加快发展县（区）域经济。落实《南宁市主体功能区规划》，引导县（区）结合各自发展潜力和优势，做强特色产业。力争横县、武鸣县、宾阳县规模以上工业总产值分别突破340亿元、240亿元、140亿元，六县地区生产总值和规模以上工业总产值对全市增长的贡献率均达27%以上；兴宁区、青秀区、西乡塘区、江南区社会消费品零售总额分别突破370亿元、345亿元、300亿元、150亿元，六城区地区生产总值和社会消费品零售总额对全市增长的贡献率达71%和75%以上。

　　加大扶贫攻坚力度。重点抓好智力扶贫，着力提升贫困群众受教育水平，提高贫困群众从事种植业、养殖业、加工业和服务业的能力，帮助农民尽快发家致富。继续抓好产业扶贫，发动5万户以上参与扶贫产业开发，发展种植类扶贫产业10万亩。修建贫困村屯级硬化路200公里以上。抓好劳动力转移就业，开辟新的增收门路，力争“十二五”期末，我市扶贫工作走在全区前列。

　　（八）以中国—东盟合作新的“钻石十年”为依托，推动对外开放合作水平新提升。

　　优化开放合作平台。积极谋划参与21世纪海上丝绸之路、泛北部湾合作和南新经济走廊建设。进一步发挥“南宁渠道”作用，创新服务面向中国和东盟合作通道建设。继续推进与国际友好城市的交流合作。密切与港澳台的开放合作。积极融入广西打造我国中南西南地区开放发展新的战略支点、珠江—西江经济带建设。主动参与南宁—崇左—凭祥对外开放经济带建设。加快实施南北钦防玉崇百区域一体化规划。

　　促进投资贸易便利化。落实出口奖励措施，扶持和组织企业参加境内外展会，鼓励企业开拓国际市场。以组织做好CEPA示范城市工作为契机，以东盟国家为主战场，鼓励企业“走出去”，参与资源开发和工程承包、劳务合作，带动商品、劳务、技术和装备出口。

　　注重招大商引强企。以港澳台、珠三角、长三角为重点，着力引进主业突出、核心竞争力强的大公司，重点落实全国工商联执委会议期间签约、洽谈的重大项目，加快对接修正药业等行业领军企业。优化招商引资环境，创新服务方式，为重大招商引资项目提供“绿色通道”。力争实际到位内资784亿元，增长8%；外商直接投资（广西全口径）6.27亿美元，增长8%。

　　继续服务好第十一届中国—东盟博览会和商务与投资峰会。完善南宁会展商务区规划，按照“扩大规模、拓展功能、丰富内容、高效便捷”的要求，加快南宁国际会展中心场馆设施和配套服务设施建设，使博览会和峰会成为建设中国—东盟自贸区升级版和21世纪海上丝绸之路的重要平台。

　　举全市之力办好第四十五届世界体操锦标赛。把办好体操世锦赛作为深化南宁对外交流与合作、扩大城市知名度、展现文明宜居城市形象的新载体。广泛动员，节俭高效、高标准、高水平做好体操世锦赛筹办和保障服务工作，并以此为契机，着力提升城市建设管理水平，提高市民文明素质，推动文化、体育、旅游、交通、环保等相关产业和城市建设有一个新的发展。

　　（九）以保障和改善民生为目的，推动社会事业全面发展。

　　全面振兴教育。牢固树立“不建楼堂建学堂”的理念，进一步加大财政投入，新建小学7所、初中4所、普通高中1所、改扩建普通高中4所，新建乡镇中心幼儿园6所、县城（城区）幼儿园2所，保障进城务工人员随迁子女入学。充分利用教育信息化，不断扩大优质教育资源覆盖面，促进义务教育均衡发展。深入实施新时期职业教育攻坚五年计划，加强校企一体化合作办学，为产业发展提供高素质技能人才。健全学生资助体系，继续在六县全面实施农村义务教育营养改善计划，对全部中职学生免学费。

　　推进就业创业工作。健全就业援助制度，深入开展“就业援助月”、“春风行动”、“高校毕业生就业服务月”和“创建充分就业县区”等系列活动。全面落实鼓励自主创业的扶持政策，推进创业孵化基地和创业服务专家队伍建设，促进自主创业。完善基层就业服务平台，扩大和完善失业监测预警，促进城镇居民收入倍增计划深入实施。

　　完善社会保障体系。扩大社会保障覆盖面，确保养老、失业、工伤、医疗、生育等保险完成自治区下达的任务，确保全市三项基本医疗保险参保（合）率稳定在97%以上。启动城乡居民大病保险工作，完善职工生育保险办法。合并新型农村社会养老保险和城镇居民社会养老保险，建立统一的城乡居民基本养老保险制度，全面实行医疗保险付费总额控制。提高企业退休人员基本养老金等社会保障待遇，做好被征地农民的社会保障工作。新开工各类保障性住房16460套，基本建成18980套，分配入住8760套，新增廉租住房租赁补贴1000户。整治改造20个无人管理的低收入群体较集中的老旧居住区。多渠道兴办养老机构，大力发展养老产业，满足不同层次的养老服务需求。加强城乡低保、五保供养、优抚安置、残疾康复、抗灾救灾等工作，完善社会救助体系。

　　着力推进文化建设。培育和践行社会主义核心价值观，努力形成良好社会风尚。加快公共文化服务体系建设，推进公共文化设施免费开放。加强非物质文化遗产保护工作。创新举办南宁国际民歌艺术节，多渠道搭建我市优秀剧（节）目对话世界的桥梁。组织开展丰富多彩的群众文化活动，扶持100支村屯社区文艺队。加快南宁市文化产业公共服务平台建设，实施动漫精品培育工程，发展南宁演艺市场产业。

　　提高人民群众健康水平。建立健全重大疾病医疗应急救助制度。巩固完善基本药物制度和基层运行新机制，行政村卫生室全部实施基本药物制度。深入推进县级公立医院综合改革。加强以社区、村卫生室和乡村医生为重点的队伍建设，完善基层医疗卫生计生服务体系，搬迁和新建江南区人民医院等4家医院。加强艾滋病等传染病防治，做好免疫规划工作。加强医院安全防范体系建设。加快中医药壮瑶医药发展。制定区域卫生规划和医疗机构设置规划，落实非公立医疗机构准入。推进市体育运动学校建设，增设一批便捷、实用公共健身设施，在人民公园等7个公园兴建7条健康步道，建设健康城市。

　　继续实施为民办实事工程。根据市人大代表、政协委员的建议，经市委常委会研究确定，将原市级20项为民办实事工程调减为10项为民办实事工程，共实施32个子项，调整后的项目更突出群众关注度高、民生关联度大、受益面广的特点。实施好自治区10项共52个子项为民办实事项目，切实办好群众期待的一批实事好事。

　　三、努力建设法治政府、服务型政府

　　加快政府职能转变，创新行政管理方式，增强政府公信力和执行力，建设法治政府和服务型政府。

　　坚持依法行政。贯彻落实《国务院关于加强法治政府建设的意见》，坚持科学决策、民主决策、依法决策，强化重大决策跟踪反馈和责任追究，提高决策质量和执行能力。加强政府立法工作，研究建立科学的法治建设指标体系和考核标准。加强政务公开，推进政社分开、政事分开和行政权力透明运行。健全行政复议案件审理机制，纠正违法或不当行政行为。依法接受市人大及其常委会监督，自觉接受人民政协的民主监督，认真听取各民主党派、工商联、无党派人士、人民团体和社会各界人士意见。充分发挥监察、审计等专门监督作用，高度重视人民群众监督和新闻舆论监督。

　　加强社会治理。改进社会治理方式，加强社区管理服务创新，激发社会组织活力。建立和完善社会稳定风险评估机制、突发事件应急处置机制。继续推进“平安南宁”建设，建立健全高效联动的立体化防控网络，依法严密防范和打击各类违法犯罪活动。启动新一轮禁毒人民战争。认真开展矛盾纠纷排查调处工作，畅通群众信访渠道，实行网上受理信访制度，健全就地解决群众合理诉求机制。深入开展安全生产隐患大检查、大整治，坚决遏制各类重特大事故发生。继续依法严厉打击“两违”。依法开展村民（社区）委员会换届选举工作。完成各级食品药品监管机构改革，深入开展专项整治行动，全力保障人民群众食品用药安全。落实国家关于调整完善计划生育的法律法规，提升全市人口长期均衡发展水平和出生人口素质。加强政府诚信、商务诚信、社会诚信和司法公信建设，构筑诚实守信的经济社会环境。

　　加强环境保护。大力发展节能环保、新能源等新兴产业，重点推进工业、建筑、交通等领域节能减排。完善环境保护考核指标体系和生态补偿机制，改革对限制开发区域和生态脆弱的国家扶贫开发工作重点县的考核机制。完成大气污染防治规划编制，加强基层环保监测执法体系建设，开展油烟、噪声、雾霾治理等专项行动，抓好城市扬尘污染、机动车排气污染、工业大气污染联防联控，环境空气质量（AQI指数）优良率保持75%以上。开展水源地综合整治，邕江地表水集中式饮用水源地水质达标率100%。

　　促进作风转变。深入开展党的群众路线教育实践活动，力戒“四风”；严格落实中央“八项规定”精神，严格遵守“约法三章”；严格控制行政经费增长，降低“三公”经费支出；树立新的政绩观，完善考评奖惩机制和激励办法；加强政风行风建设，纠正损害群众利益的不正之风；深入推进惩治和预防腐败体系建设，严肃查处违纪违法案件，做到干部清正、政府清廉、政治清明。

　　全面推进全国民族团结进步示范市建设，巩固和发展平等团结互助和谐社会主义民族关系。加强国防动员和后备建设力量，支持国防和军队建设，广泛开展双拥共建活动，推动军民融合深度发展。依法保障公民特别是妇女、未成年人和残疾人的合法权益。充分发挥工会、共青团、妇联等群团组织的作用。继续做好外事、侨务、宗教、统计、人防、气象、地震、水文、供销、参事、档案等工作。

　　各位代表，新的一年，任务艰巨、使命光荣、责任重大。让我们紧密团结在以习近平同志为总书记的党中央周围，在自治区党委、政府和市委的正确领导下，齐心协力，开拓创新，勤勉工作，奋力提升首府南宁在广西经济社会发展中的首位度，为加快建设中国面向东盟开放合作的区域性国际城市、宜居的壮乡首府和具有亚热带风情的生态园林城市而努力奋斗！
